# Veneral Prophylaxis Post Hoc

**Published:** 1913-04

**Authors:** 


Veneral Prophylaxis Post Hoc.—Col. L. Marvin Maus, U. S. A., *Prov. Med. Jour.*, Jan., 1913, in a general article, includes interesting statistics as to chemic prophylaxis after exposure. The fact that a considerable number of exposures were deliberately experimental, will delight the antivisectionists, who claim that animal experimentation is the indication of a species of perversion whose acme is attained by experiment on the human being. The following table shows the results of the author on sixteen volunteers, shown to be gonococcus-free, inoculated with pus shsown to contain gonococci:

| Group | Formula | Time after Inoculation | Results |
|---|---|---|---|
| 1. | Argyrol, 20%; calomel, 33%; fatty base of ointment, 47%....... | 10 minutes | Successful |
| 2. | Protargal, 2%; calomel, 33%; fatty base of ointment, 65%....... | 15 minutes | Successful |
| 3. | Thymol, 5%; calomel, 33%; fatty base of ointment, 62%....... | 20 minutes | Successful |
| 4. | Phenol, 3%; calomel, 33 %; fatty base of ointment, 64%....... | 20 minutes | Successful |
| 5. | Hyco, 2%; calomel, 33%; fatty base of ointment, 65%........... | 25 minutes | Successful |
| 6. | Calomel, 33%; fatty base of ointment, 67%.................. | 25 minutes | Successful |
| 7. | Calomel, 25%; fatty base of ointment, 75%.................. | 30 minutes | Successful |
| 8. | Calomel, 20%; fatty base of ointment, 67½.................. | 25 minutes | Successful |

Dr. Gustave M. Blech of Chicago confirmed my experiments on sixteen volunteer Greeks and Italians at the time patients at Lincoln Hospital in that city. He employed the same technique in regard to the examinations of urethal scrapings and gonorrheal pus in order to satisfy himself tthat the men were free from infection at date of experiment and that the pus used in the inoculations was gonorrheal.

Collapsible tubes filled with 4 c. c. of calomel ointment (1-3 strength) were issued to 1200 regulars at a military tournament at Chicago. No new cases developed among those using the prophylactic. In September, 1910, a similar observation was carried out, 594 men, with 1301 exposures, using the prophylactic developed no new infection. Among 302 men, with 763 exposures, who neglected the prophylactic, there developed 26 cases of gonorrhoea, 12 of chancroid and 1 of syphilis. Various other corroboratory notes are added. (Note.—We believe nobody will accuse us of being an anti—or order his subscription stopped, if we state plainly that we do not approve of deliberate experiment of this sort on human beings. The fact that the subjects of experiment were volunteers does not alter this view. One of the cardinal functions of the medical profession is to avoid risks and to protect human beings against their own lack of caution. Of course, the successful result of the experiment affords very valuable and scientific information, but of equal value would be a failure, and we doubt very much whether investigators would dare to report unsuccessful cases.—Ed.)

